# When Phenotype Outspeaks Genotype: Uncommon Vascular Anomalies in Suspected Hereditary Hemorrhagic Telangiectasia Despite Negative Genetic Testing

**DOI:** 10.7759/cureus.109941

**Published:** 2026-05-30

**Authors:** Nayef Alkhalil, Estello Nap-Hill, Benjamin Cox, Eric M Yoshida, Daljeet Chahal, Vladimir Marquez-Azalgara

**Affiliations:** 1 Division of Gastroenterology and Hepatology, Department of Medicine, University of British Columbia, Vancouver, CAN; 2 Division of General Internal Medicine, Department of Medicine, University of British Columbia, Vancouver, CAN

**Keywords:** ehlers-danlos syndrome, hereditary hemorrhagic telangiectasia, negative genetic testing, osler-weber-rendu syndrome, rare vascular disorder, vascular malformations, visceral arteriovenous malformations

## Abstract

Hereditary hemorrhagic telangiectasia (HHT) is an inherited vascular disorder characterized by abnormal blood vessel formation involving the skin and visceral organs. Establishing the diagnosis can be challenging, particularly in the absence of classic clinical manifestations or confirmatory genetic findings. We report the case of a 42-year-old woman who presented with acute epigastric pain and was found to have extensive vascular abnormalities involving the liver, lungs, aorta, and mesenteric circulation. She had no history of recurrent epistaxis, mucocutaneous telangiectasias, or family history of vascular disease, and genetic testing for known vascular disorders was negative. Despite the absence of typical clinical and genetic features, the distribution and extent of vascular involvement raised a strong suspicion for underlying HHT. This case highlights the potential for a broader phenotypic spectrum of HHT and underscores the limitations of current diagnostic criteria and genetic testing approaches.

## Introduction

Hereditary hemorrhagic telangiectasia (HHT), also known as Osler-Weber-Rendu syndrome, is an autosomal dominant vascular disorder characterized by mucocutaneous telangiectasias and arteriovenous malformations (AVMs) involving visceral organs such as the liver, lungs, and brain. It has an estimated prevalence of approximately one in 5,000 to 8,000 individuals [1]. Diagnosis is primarily based on the Curaçao criteria and is often supported by genetic testing for pathogenic variants in key genes involved in vascular signaling pathways [2].

At the molecular level, HHT results from loss-of-function mutations in genes encoding components of the transforming growth factor-beta (TGF-β)/bone morphogenetic protein (BMP) signaling pathway, which plays a central role in vascular development and endothelial cell homeostasis [1,3]. The majority of cases are attributable to pathogenic variants in three genes: ENG (9q34.11), ACVRL1 (12q13.13), and SMAD4 (encoding Mothers Against Decapentaplegic Homolog 4). Together, ENG and ACVRL1 account for up to 90% of disease-causing variants, while SMAD4 mutations are responsible for approximately 1%-2% of cases [1,2]. However, despite advances in genetic testing, approximately 10% of clinically suspected cases remain without an identifiable pathogenic variant in known HHT-associated genes [2,4].

The liver is among the most frequently affected visceral organs in HHT, with liver vascular malformations (LVMs) detected on imaging in up to 70% of patients with definite disease. The presence of LVMs is associated with increased morbidity and mortality, and clinically significant disease occurs more commonly in females [5,6].

This case highlights an atypical presentation of suspected HHT in a female patient with multiple visceral and arterial vascular anomalies, despite fulfilling only one Curaçao criterion and having negative genetic testing.

This case report was previously presented as a poster presentation at the Joint Canadian Digestive Diseases Week and Canadian Liver Meeting (CDDW-CLM) Conference 2026 in Toronto, Canada.

## Case presentation

A 42-year-old woman of Filipino origin with no significant past medical history presented with 24 hours of sharp, non-radiating epigastric pain, nausea, and blood-tinged emesis. On admission, she was hypertensive with systolic blood pressure ranging from 180 to 190 mmHg but remained hemodynamically stable. Abdominal examination demonstrated localized epigastric and right upper quadrant tenderness without peritoneal signs. No mucocutaneous telangiectasias, cutaneous lesions, or clinical stigmata of chronic liver disease were identified.

Initial laboratory investigations revealed leukocytosis (WBC 12.2 × 10⁹/L), elevated transaminases, mild hyperbilirubinemia, and an elevated C-reactive protein. Beta human chorionic gonadotropin (β-hCG) was negative, and lipase was within normal limits. Table 1 presents a complete listing of the laboratory results.

**Table 1:** Patient’s initial laboratory results WBCs: white blood cells; ALT: alanine transaminase; AST: aspartate aminotransferase; GGT: gamma-glutamyl transpeptidase; ALP: alkaline phosphatase; β-HCG: beta human chorionic gonadotropin, CRP: C-reactive protein

Lab Test	Result	Reference Range
WBCs	12.2	4.0-10.5 × 10⁹/L
Hemoglobin	164	120-150 g/L
Platelets	170	150-400 × 10⁹/L
Creatinine	47	49-90 umol/L
Sodium	139	135-145 mmol/L
Potassium	3.3	3.5-5 mmol/L
Chloride	102	98-107 mmol/L
Bicarbonate	26	20-31 mmol/L
ALT	319	< 45 U/L
AST	210	13-40 U/L
GGT	41	< 38 U/L
ALP	69	46-116 U/L
Bilirubin Total	33	5-21 umol/L
Lipase	32	12-53 U/L
β-HCG	Negative	Negative
CRP	136.2	< 5 mg/L

Abdominal ultrasound demonstrated a heterogeneous liver parenchyma with prominent vascular structures at the porta hepatis (Figure 1). Contrast-enhanced CT of the abdomen and pelvis showed multiple dilated intrahepatic vascular communications, predominantly involving segments IV, VI, and VII, consistent with intrahepatic portovenous shunting (Figure 2).

**Figure 1:**
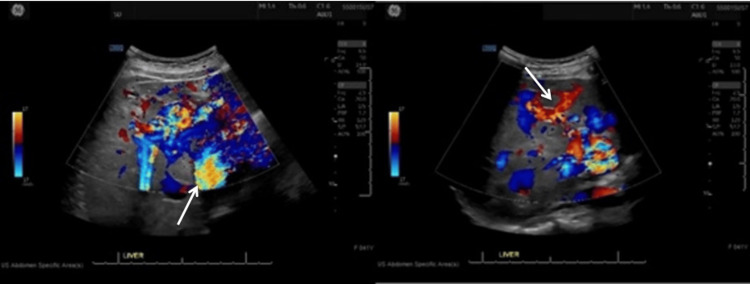
Doppler abdominal ultrasound images of the liver demonstrating prominent intrahepatic vascular communications (white arrows).

**Figure 2:**
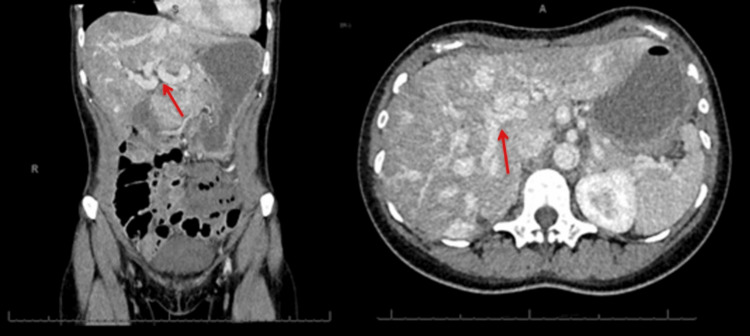
Coronal and axial contrast-enhanced CT Images of the abdomen showing intrahepatic arteriovenous malformations (red arrows).

Further multiphasic thoracoabdominal imaging revealed multiple systemic vascular abnormalities, including pulmonary AVMs (Figure 3), a chronic type B aortic dissection extending from the thoracolumbar region to the infrarenal aorta, and occlusion of two large proximal branches of the superior mesenteric artery. Brain imaging showed no evidence of cerebral AVMs.

**Figure 3:**
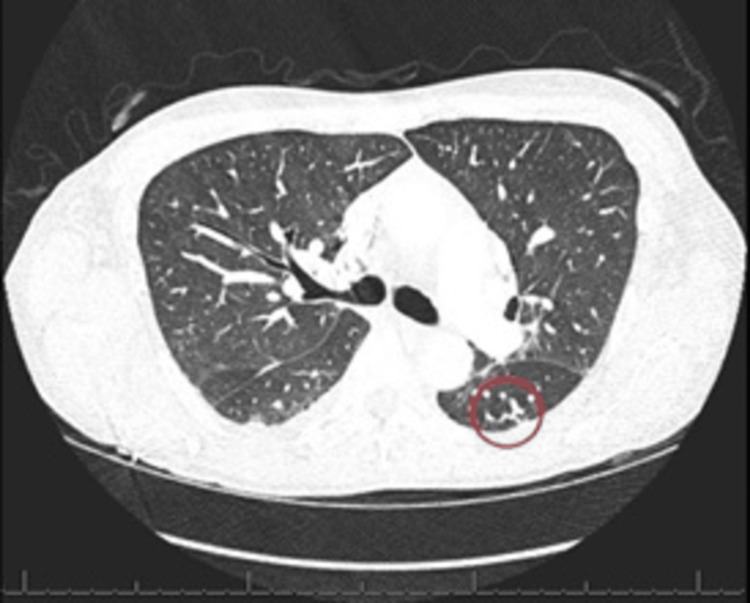
Axial CT image of the chest demonstrating a pulmonary arteriovenous malformation in the left lower lobe (red circle).

Given the coexistence of pulmonary and hepatic AVMs, underlying HHT was suspected despite the absence of epistaxis, mucocutaneous telangiectasias, or family history. Genetic testing for HHT and connective tissue disorders, including Ehlers-Danlos syndrome, was negative. The patient was clinically stabilized and discharged.

She was subsequently readmitted with recurrent symptoms. MRI and MR cholangiopancreatography demonstrated mild intrahepatic biliary dilatation with beading and strictures, consistent with ischemic cholangiopathy, as well as a 6 cm biloma involving segments VII and VIII (Figure 4). Liver elastography showed a stiffness of 5.58 kPa, consistent with no significant fibrosis. Workup for chronic liver disease, including viral, autoimmune, and genetic causes, was negative. Upper endoscopy performed for evaluation of epigastric pain revealed mild esophagitis (Grade A as per Los Angeles classification), a clean-based duodenal ulcer at the D1-D2 junction, and multiple small antral gastric ulcers.

**Figure 4:**
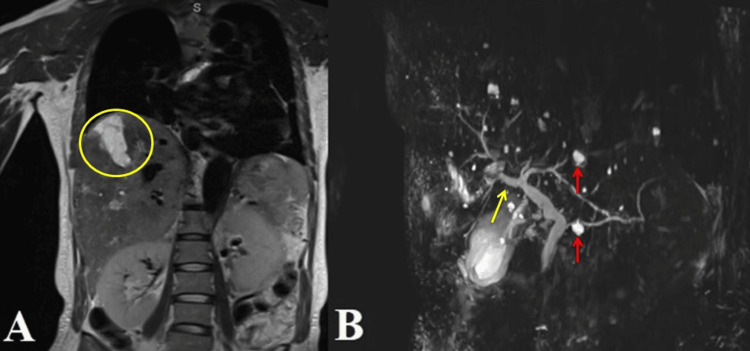
Coronal T2-weighted MRI image of the abdomen (A) and heavily T2-weighted MRCP image (B) showing a biloma (yellow circle) with associated prominent bile ducts (yellow arrow) and biliary cysts (red arrows). MRCP: magnetic resonance cholangiopancreatography

In the setting of progressive cholangiopathy and worsening liver function, she was evaluated for liver transplantation. A multidisciplinary consensus recommended embolization of her pulmonary AVMs prior to transplant listing. However, she was found deceased at home a few days following pulmonary AVM embolization. The cause of death remained undetermined, as the family declined an autopsy.

## Discussion

HHT is an uncommon vascular dysplasia characterized by fragile mucocutaneous telangiectasias and visceral AVMs that cause chronic gastrointestinal bleeding, iron-deficiency anemia, and organ-specific complications such as stroke, high-output heart failure, and liver disease [1]. Despite its substantial morbidity and potential for reduced overall survival, HHT remains widely underdiagnosed, with a mean interval between first symptoms and diagnosis exceeding two decades [7].

HHT is caused by a loss-of-function pathogenic mutation in genes encoding components of the BMP/TGF-β signaling pathway, which is critical for vascular development and endothelial cell homeostasis [1,3].

HHT is diagnosed clinically using the Curacao criteria, which include: (1) spontaneous, recurrent epistaxis; (2) multiple mucocutaneous telangiectasias at characteristic sites (lips, oral cavity, fingers, nose); (3) visceral AVMs (e.g., pulmonary, cerebral, hepatic, gastrointestinal); and (4) a first-degree relative with HHT. A diagnosis is considered definite if three or more criteria are met, possible if only two are met, and unlikely if fewer than two are present [1,2,4]. Our patient met only one criterion, namely visceral AVMs involving the liver and lungs, and is therefore considered unlikely to have HHT according to the Curaçao criteria.

In patients who meet definite clinical criteria for the Curaçao criteria, defined as three or more clinical features, pathogenic variants in the major genes associated with HHT (ENG, ACVRL1, and SMAD4) are identified in more than 90% of cases. Despite this high diagnostic yield, approximately 4%-10% of patients with clinically definite disease remain without an identifiable mutation [2,8,9].

When genetic testing is applied to broader and more heterogeneous populations, including individuals with possible or suspected HHT based on fewer clinical criteria or atypical presentations, the overall diagnostic yield decreases to approximately 85%. Consequently, up to 15% of tested individuals may have negative genetic results despite clinical suspicion of HHT [8,10,11]. This reduction in yield is likely related to the lower pretest probability of true HHT in patients who do not meet full clinical criteria [2,12]. In addition, emerging evidence suggests that a subset of genetically unexplained cases may be attributable to as-yet-unidentified genes involved in the HHT pathway [9].

Our patient demonstrates an atypical vascular phenotype. Although she is classified as unlikely for HHT as per traditional standards, as previously discussed, the coexistence of pulmonary and intrahepatic AVMs is highly suggestive of an underlying HHT spectrum disorder.

Based on the available literature, no single alternative disease process has been reported to account for the coexistence of both pulmonary and hepatic AVMs. HHT accounts for up to 90% of pulmonary AVMs, and pulmonary AVMs may occasionally represent the sole clinical manifestation in otherwise confirmed cases of HHT [13,14]. Alternative explanations would therefore require the presence of two independent vascular pathologies occurring concurrently, which is considered unlikely given the extent and systemic distribution of vascular involvement.

In addition, it is plausible to believe that the patient carries an unknown genetic mutation that may fall outside the scope of existing HHT genetic panels. Novel candidate genes have been proposed as putative drivers in patients with HHT phenotypes who test negative for standard mutations [9].

This case is notable for the coexistence of extensive arterial anomalies, including chronic type B aortic dissection and large mesenteric arterial occlusions, features not typically associated with HHT [15]. This prompted consideration of vascular connective tissue disorders such as vascular Ehlers-Danlos syndrome (vEDS) in the differential diagnosis. However, the absence of typical clinical features (e.g., thin, translucent skin, easy bruising, characteristic facial features, and small-joint hypermobility) and the negative genetic panel results argue against vEDS [16].

The biliary pathologies in this case, ischemic cholangiopathy and biloma, are sequelae of vascular steal syndrome secondary to hepatic AVMs, a known complication in HHT [5,6,17]. Hepatic artery-to-hepatic vein or hepatic artery-to-portal vein shunting can cause biliary ischemia due to reduced arterial perfusion of the bile ducts, which are exclusively supplied by the hepatic artery. This can lead to biliary necrosis, biloma formation (which may become infected), and secondary sclerosing cholangitis [5,16]. Importantly, no significant hepatic fibrosis was found, supporting the conclusion that the biliary changes were vascular in origin rather than related to chronic liver disease. Liver synthetic function typically remains normal in HHT-related liver vascular malformations unless advanced secondary biliary cirrhosis develops [5]. 

This case broadens our understanding of HHT and its clinical spectrum by demonstrating that significant arterial abnormalities can be present in patients with limited or atypical clinical features. It also underscores the limitations of relying solely on conventional diagnostic tests in cases with compelling radiologic evidence of systemic vascular pathology.

## Conclusions

This case illustrates a rare and atypical presentation of a possible HHT spectrum disorder in a genetically negative patient with only one diagnostic criterion. The presence of pulmonary AVMs, intrahepatic shunts, chronic aortic dissection, and mesenteric arterial occlusions suggests a broader vascular dysplasia phenotype that current HHT criteria may not fully encompass. Although the clinical and radiologic findings raise a strong suspicion for HHT or a related vascular disorder, the diagnosis remains presumptive in the absence of molecular confirmation. Clinicians should maintain a high index of suspicion for HHT even in the absence of classical features or positive genetic testing, while recognizing the limitations of current diagnostic criteria and genetic panels.
